# Is the unique camouflage strategy of *Pneumocystis* associated with its particular niche within host lungs?

**DOI:** 10.1371/journal.ppat.1007480

**Published:** 2019-01-24

**Authors:** Philippe M. Hauser

**Affiliations:** Institute of Microbiology, Lausanne University Hospital and University of Lausanne, Lausanne, Switzerland; McGill University, CANADA

The fungal genus *Pneumocystis* includes species that colonize mammalian lungs. If the immune system of the host weakens, these obligate parasites can turn into opportunistic pathogens causing deadly pneumonia. Each *Pneumocystis* species presents a strict specificity for a single mammalian species, although few exceptions may exist in rodents [1]. The lack of an established long-term method of in vitro cultivation for these fungi has considerably hindered their study. Nevertheless, the advent of high throughput methods allowed sequencing the genomes of *Pneumocystis jirovecii*, *P*. *carinii*, and *P*. *murina*, infecting, respectively, humans, rats, and mice [2,3]. The size of these genomes is approximately 8 Mb, their guanine-cytosine content is approximately 28%, and their number of chromosomes is approximately 17. However, the telomeres, subtelomeres, and centromeres remained elusive because their repetitive nature prevented assembling them. The subtelomeres of *Pneumocystis* species harbor genes encoding glycoproteins that are believed to be responsible for important virulence factors, i.e., surface antigenic variation and adhesion to host tissues [4–6]. Surface antigenic variation is thought to allow escape from the host immunity during colonization, and approximately 5% of each *Pneumocystis* genome is dedicated to this system. Antigenic variation is a common strategy among microbial mammalian pathogens. The systems often rely on gene families encoding surface antigens localized at subtelomeres; presumably because these genomic regions are prone to gene silencing and perhaps enhanced mutagenesis [7]. Moreover, the clusters of telomeres that are formed at the nuclear periphery during meiosis may favor ectopic recombinations, which can be responsible for the generation of new mosaic antigens [8]. The advent of a DNA sequencing method generating long reads recently allowed assembly of *Pneumocystis* subtelomeres and characterization of their gene content. This revealed their organization and new gene families encoding surface glycoproteins that constitute a superfamily. In this review, I update the understanding of the system and the strategy of antigenic variation of *Pneumocystis* species in the light of these new observations.

## What does the major surface glycoproteins superfamily consist of?

A total of eight different gene families encoding major surface glycoproteins (Msgs) were identified in *Pneumocystis* species [3,9]. Their relevant features are given in Table 1. They are localized at the subtelomeres, except family C that forms an intrachromosomal tandem cluster in *P*. *murina*. Each gene family includes 1 to 80 of similar genes per genome. Phylogenetic analyses showed that these families are related and thus form a superfamily. Only six Msg families are present in *P*. *jirovecii* and *P*. *carinii*, whereas *P*. *murina* harbors seven of them. Families IV and C are present only in, respectively, *P*. *jirovecii* and *P*. *murina*, whereas family Msg-related (Msr) is harbored only by *P*. *carinii* and *P*. *murina* [3,10]. The structure of the genes and proteins of these families are described in the next sections.

**Table 1:** Features of the eight gene families constituting the Msg superfamily identified in *Pneumocystis* species.

Msg family name [9]	Other Msg family name [3]	Approximate gene size (bps)	Gene expression mode	Gene location in subtelomeres relative to telomere	Percent mosaic genes in *P*. *jirovecii* [9]^b^	Percent total Msg genes (total genes observed) in *P*. *jirovecii* (74)^c^ [9]	Percent total Msg genes (total genes observed) in *P*. *jirovecii* (177) [3]	Percent total Msg genes (total genes observed) in *P*. *carinii* (140) [3]	Percent total Msg genes (total genes observed) in *P*. *murina* (64) [3]
I	A1	3100	mutually exclusive^d^	proximal	42	35	45	47	39
Msr	A2	3100	independent	central	NA	0	0	36	22
II	A3^f^	3100	independent	central	28	19	29	12	17
III					40	12			
IV	B	2000	independent	central	22	8	12	0	0
NA	C^g^	1500	Independent	Intrachromosomal tandem cluster	NA	0	0	0	6
V	D	3100	independent	central	7	18	11	1	2
VI	E	1200	independent	distal	0	8	3	4	11

^a^Family C is the only family not localized at the subtelomeres.

^b^No data are available for *P*. *carinii* and *P*. *murina*.

^c^The partial genes due to their truncation by the end of the contig were taken into account.

^d^A single isoform is expressed under the control of the transcription promoter of the UCS that is present at a single copy per genome.

^e^A transcription promoter is present upstream of each gene, suggesting independent expression.

^f^Family A3 corresponds to both families Msg-II and -III (see Figure S11 of reference [9]).

^g^The two and one *msg*-C genes reported, respectively, in *P*. *jirovecii* and *P*. *carinii* [3] are not included here. Indeed, those of *P*. *jirovecii* were shown to correspond to pseudogenes of Family I [9], and that of *P*. *carinii* was small (213 amino acids) and thus potentially a gene fragment or also a pseudogene.

**Abbreviations:** Msr, Msg-related; Msg, major surface glycoprotein; NA, not applicable; UCS, upstream conserved element.

## What is the structure of the genes belonging to the different Msg families?

**Fig 1:**
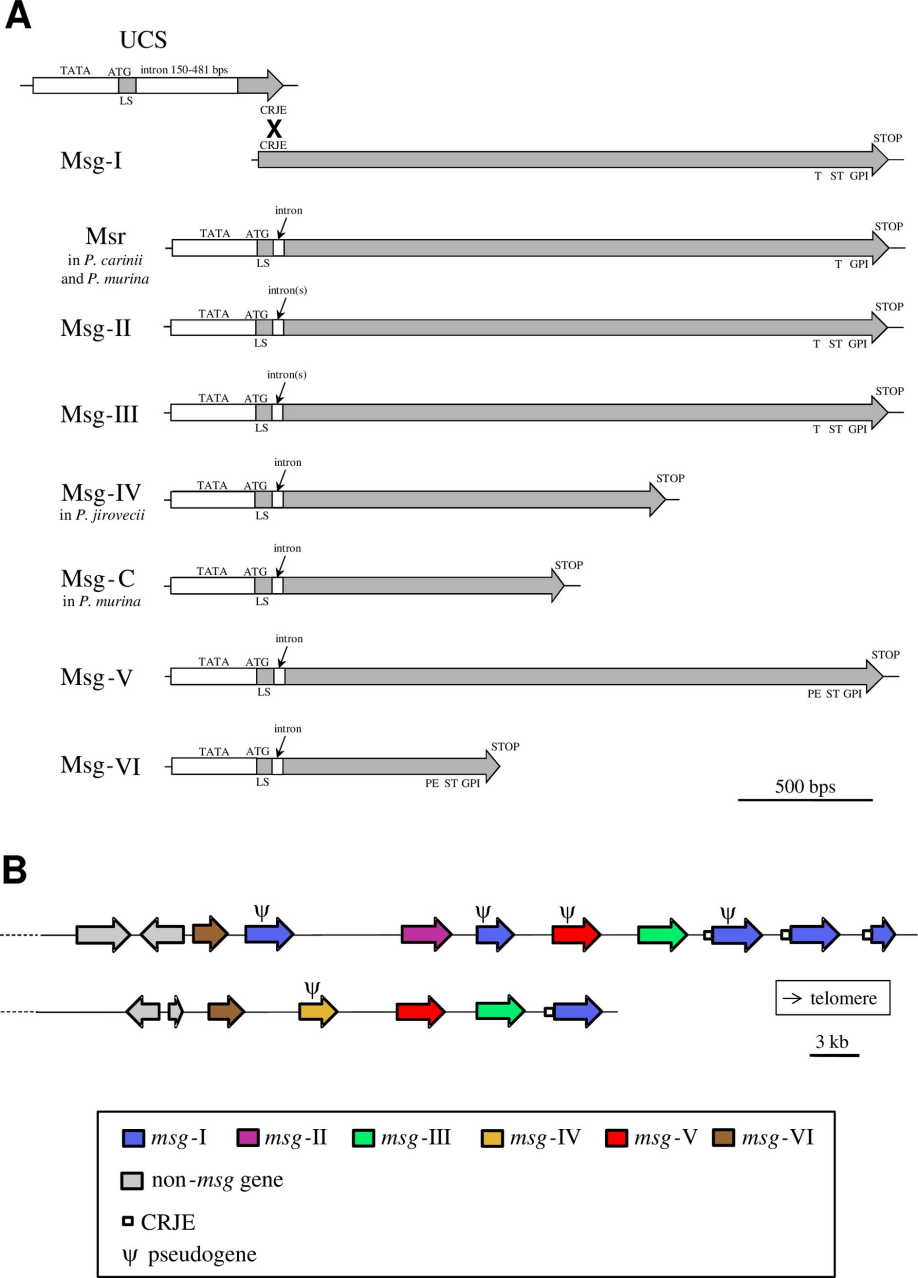
Structure and organization of the Msg superfamily of *Pneumocystis* species. (A) The gene structure of each Msg family in shown with underneath features of the encoded glycoprotein. For Family I, the cross figures recombination between the CRJE sequences involved in mutually exclusive expression of a single gene. In *Pneumocystis jirovecii*, there are two introns instead of one in Families II and III. Except in Family I, each intron is 20 to 50 bps long. (B) Organization of the *msg* genes within typical subtelomeres of *P*. *jirovecii*. These subtelomeres are from chromosome 6 (top) and 11 (bottom) [9]. CRJE, conserved recombination junction element; GPI, glycosylphosphatidylinositol-anchor signal; LS, leader sequence; Msg, major surface glycoprotein; PE, proline and glutamine-rich region; ST, serine and threonine-rich region; T, threonine-rich region; UCS, upstream conserved element.

These structures are shown in panel A of Fig 1. Each *Pneumocystis* cell would express a single gene of Family I thanks to its localization downstream of a subtelomeric expression site, the upstream conserved element (UCS), which is present at a single copy in the genome (a system called “mutually exclusive expression”). In contrast, each gene of all families possesses its own upstream promoter and protein start, suggesting an independent expression rather than mutually exclusive expression. The UCS encompasses a promoter of transcription that has strong activity, which is consistent with the fact that the single isoform of Family I produced is the most abundant at the cell surface [11]. Exchange of the expressed *msg* gene would rely on recombination at a short sequence that is present both at the end of UCS and at the beginning of each *msg* gene (the conserved recombination junction element [CRJE]; Fig 1A, Family Msg-I). The CRJE is 33, 28, and 132 bps long in, respectively, *P*. *jirovecii*, *P*. *carinii*, and *P*. *murina*. All genes of all families, including that of Family I linked to the UCS, present one or two introns only at their 5ʹ end. The localization of the genes of each family within typical *P*. *jirovecii* subtelomeres is shown in panel B of Fig 1. Genes of Family I are closest to the telomere, those of Family VI closest to the non-*msg* genes, and those of the other families locate centrally within the subtelomeres. Identical localizations were reported within the subtelomeres of *P*. *carinii* and *P*. *murina* [3].

## What is the structure of the proteins belonging to the different Msg families?

These structures are also shown in panel A of Fig 1, underneath the gene structures. All proteins present a sequence leader suggesting their translocation into the endoplasmic reticulum. The presence of a glycosylphosphatidylinositol (GPI)-anchor signal for all families, except Families IV and C, suggests that these proteins end up attached to the cell membrane or wall. Surface localization has been assessed by antibodies raised against (i) purified Msg proteins that probably contained members of Families I, II, III, V because these are all GPI-anchored and of similar size, and (ii) *P*. *murina* proteins of Family VI [12]. Proteins of Families IV and C that are specific to, respectively, *P*. *jirovecii* and *P*. *murina* lack a GPI-anchor signal, suggesting that they might be excreted outside of the cell. The CRJE sequence of Family I encodes a potential lysine-arginine recognition site that might be involved in the release of the constant part of the antigen. In *P*. *jirovecii* and *P*. *murina*, the Kexin endoprotease potentially responsible for this maturation is encoded by a single gene and is localized in the Golgi apparatus [13,14]. In contrast, in *P*. *carinii*, several Kexin endoproteases are encoded by a subtelomeric family including approximately 15 genes and are present at the cell surface [15].

## How would antigenic variation be generated?

Exchange of the gene of Family I expressed under the control of the promoter present in the UCS by recombination at the CRJE sequences would constitute a first mechanism of antigenic variation. The localization of the *msg*-I genes closest to the telomere might favor this exchange because it may facilitate the exchange of the telomeres that is required by a single recombination at the CRJE sequences (see panel B of Fig 1 and Fig 5 of reference [9]). Studies in *P*. *carinii* suggested that the maximum rate of switching the Msg I isoform expressed is 0.01 event per generation [16]. Consistently, a number of different expressed genes were identified linked to the UCS in each *P*. *jirovecii* isolate from a single patient [17], up to 18 [9]. A second mechanism of antigenic variation would be the frequent homologous recombinations that were shown to occur between the isoforms of family I [18]. Using bioinformatics tools, signatures of recombination events were recently detected in all *P*. *jirovecii* Msg families, except in Family VI, and substantial proportions of the genes were putative mosaics (up to 42%, Table 1). No recombinations were detected between members of different Msg families, suggesting that the families diverged sufficiently to prevent such events. Identities among Msg genes between 45% and 66% were suggested to be the lowest level of similarity allowing homologous recombination [9]. Because recombinations might occur sometimes between poorly homologous sequences, they might break the open reading frames present in Msg genes. Such events might contribute to the birth of the pseudogenes that have been identified in all *P*. *jirovecii* Msg families, except in Family VI (Fig 1, panel B) [9]. Nevertheless, the supposedly increased mutagenesis rate and genetic drift within the subtelomeres might also be involved in the birth of these pseudogenes. On the other hand, purifying selection and homologous recombination might contribute to the removal of the deleterious mutations within pseudogenes. The absence of selection on the nonfunctional Msg genes may also explain a high rate of pseudogenes.

## What are the functions of the different Msgs

*Pneumocystis* Msgs are potentially responsible for escaping from the host immune system through their variation by mosaicism and mutually exclusive expression of Family I. This role is strongly suggested by the facts (i) that systems involving mutually exclusive expression and variation through gene mosaicism are also active in *Plasmodium* and *Trypanosoma* for which immune escape is established, and (ii) that the host immune system exerts a selective pressure on the evolution of *Pneumocystis* Msgs and several proteins involved in GPI biosynthesis [19]. These two latter classes of proteins showed an accelerated evolution consistent with their implication in the interactions with the host. In addition, Msgs are probably involved in adhesion to host cells [20–22]. Indeed, they are all made of one to five Msg domains specific to the *Pneumocystis* genus that are made of approximately 75 residues presenting regularly separated conserved cysteines (Pfam PF02349) [3,9]. Such structure is similar to leucine zipper motifs that are known to be involved in nonspecific protein–protein interaction. Moreover, Msgs have been demonstrated to be involved in adhesion to (i) constituents of the extracellular matrix present between lung epithelial cells (fibronectin, vibronectin, laminin) [20], (ii) lung surfactant protein D [21], and (iii) macrophage mannose receptors [22]. Consistently, all Msgs, except those of Families IV and C, were predicted to be fungal adhesins using a bioinformatics tool based on signatures conserved among fungal adhesins [23], and all present sites of nitrogen-linked glycosylation that are known to be crucial in pathogen–host interactions. Moreover, they fit the model of fungal adhesin structure with a serine and threonine-rich region at the C terminus responsible for stiffening of the molecule through O-glycosylation and a ligand-binding domain at the N terminus [24]. The localization and function(s) of Families IV and C remain to be determined since they have no GPI-anchor signal as well as no serine and threonine-rich region. Recently, *P*. *murina* Msg-VI glycoproteins have been shown to be present at the surface of ascospores within asci [12]. This suggests that the Msg families are differentially expressed during the life cycle.

## How does the Msg superfamily vary among the different *Pneumocystis* species?

The proportions of the different Msgs observed in the three *Pneumocystis* species are given in Table 1. In addition to the lack of one or two Msg families in each *Pneumocystis* species, *P*. *jirovecii* present (i) a clear extension of Family V and (ii) a slight extension of Families II and III relative to the other species. The variation of the set of Msg families might reflect the characteristics of the specific niche to which the *Pneumocystis* species has adapted. Accordingly, the fact that the Msg sets of *P*. *carinii* and *P*. *murina* are similar, except the additional presence of Family C in *P*. *murina*, might reflect that they both infect rodents. One can hypothesize that the composition of each Msg set is involved in the host specificity of the *Pneumocystis* species. Each Msg set might confer the ability to adhere to specific host tissues and escape from the specific host immune system present in the niche.

## What is the *Pneumocystis* cell surface structure?

The surface of *Pneumocystis* cells is likely to include a mixture of the different Msgs, with a majority of those of Family I. In addition, it would include at least the other surface proteins that have been reported [25,26], and, in the case of *P*. *carinii*, the Kexin endoproteases. The conserved cysteines of the Msg domains, as well as the proline and glutamine-rich regions present in Families V and VI, are known to be involved in nonspecific protein–protein interaction, as well as in dimerization. Msgs also present coiled-coil domains that are often involved in the formation of heteromultimers and protein complexes [9]. These observations suggest that the various *Pneumocystis* surface proteins might form homo- and/or hetero-oligomers resulting in a dense coat. Consistently, Msgs were previously reported to form multimers [27]. Obviously, deciphering the complex structure of *Pneumocystis* cell surface will require further research.

## Why such a unique strategy of camouflage?

In the immunocompromised host, *Pneumocystis* species seem to continuously produce cells expressing a new Msg I isoform as well as new mosaic Msgs of the other families. This strategy of antigenic variation generates cell populations made of subpopulations that are antigenically different. In the colonized host, i.e., where the *Pneumocystis* system of antigenic variation evolved, there might be less numerous subpopulations, or even a single one at a time, because of the selection by the host immune response. However, this has not been studied so far. The occurrence of genetic mosaicism is further suggested by (i) the important variability of the subtelomeres between *P*. *jirovecii* isolates that is compatible with frequent recombinations within these regions [3], and (ii) the obligate nature of *Pneumocystis* sexuality because ectopic recombinations between subtelomeres might occur mostly during meiosis [28] within the cluster of telomeres that forms in eukaryotes to promote pairing of homologous chromosomes [29]. Such a strategy of antigenic variation involving mutually exclusive expression together with continuous expression of various mosaic antigens appears unique among pathogens. It might be associated with the particular niche within mammalian lungs. Indeed, bacteria and fungal spores enter into the lungs constantly at each breath. Therefore, a small amount of microorganisms is always present within the lungs, which stimulates the host immune system. The constant activity of the immune system in the lungs might force *Pneumocystis* species presenting most cells of their population as different antigenically. This might mimic the presence of microorganisms at low abundance and so allow being tolerated by the immune system. The strategy to present continuously new antigenically different cells corresponds to an adaptation from “standing genetic variation,” i.e., the continuous presence of several alleles at a locus in a population [30]. In contrast, pathogens whose niche is host blood or tissue, such as *Plasmodium* and *Trypanosoma*, face a strong immune reaction directed specifically against them because no microorganisms are tolerated in these sterile niches. Their strategy is to produce cell populations that are homogenous antigenically until the single expressed surface antigen is recognized by the immune system [31]. Subsequently, switching of the antigen isoform expressed occurs, permitting regrowth of the parasite. A single gene is expressed at a time thanks to a tight control of the mutually exclusive expression, avoiding exposure of the antigenic repertoire present in their genome. This strategy drastically contrasts with that of *Pneumocystis*, which would rely on expression of many surface antigens simultaneously in each population. The strategy of *Pneumocystis* also differs from those used by *Candida* spp. that inhabit other nonsterile niches of the human body (skin, gut, vagina). Indeed, *Candida glabrata* relies on epigenetic silencing of its adhesin gene family with induction of expression by external stimuli [32]. *C*. *albicans* uses differential expression of its adhesin genes in yeast or hyphae with codon mistranslation to produce surface variation [33].

## Conclusion

The question raised in the title of the present review cannot be presently answered but constitutes a valid working hypothesis: the unique strategy of antigenic variation used by *Pneumocystis* may allow survival within the nonsterile mammalian lungs. Crucial questions concerning *Pneumocystis* antigenic variation still pend: how is the expression of the *msg* genes presenting their own promoter regulated? Is gene silencing involved? What are the function(s) of the different Msg families? Where are their ligand-domains? Do the Msgs have other functions than adhesion? Do the Kexin endoproteases act on all Msg families? Do the *P*. *carinii* Kexin endoproteases contribute to antigenic variation by other means than removal of the UCS? Do all Msg families elicit host immune response? Does the likely absence of hyper-mannan glycosylation of Msg [3] contribute to evasion from the host immune system? What is the frequency of switching of the expressed gene of Family I in each *Pneumocystis* species? Is telomere exchange involved in this switching rather than gene conversion? What is the frequency of the recombinations creating mosaic genes within each Msg family? Are these recombinations evenly distributed over the length of Msg genes? Are there further proteins involved in the structure of *Pneumocystis* surface?
